# Association of TrkA and APP Is Promoted by NGF and Reduced by Cell Death-Promoting Agents

**DOI:** 10.3389/fnmol.2017.00015

**Published:** 2017-01-31

**Authors:** Nadia Canu, Ilaria Pagano, Luca Rosario La Rosa, Marsha Pellegrino, Maria Teresa Ciotti, Delio Mercanti, Fabiola Moretti, Valentina Sposato, Viviana Triaca, Carla Petrella, Ichiro N. Maruyama, Andrea Levi, Pietro Calissano

**Affiliations:** ^1^Department of System Medicine, University of Rome “Tor Vergata”Rome, Italy; ^2^Institute of Cellular Biology and Neurobiology, National Council of Research of RomeRome, Italy; ^3^European Brain Research InstituteRome, Italy; ^4^Information Processing Biology Unit, Okinawa Institute of Science and Technology Graduate UniversityOkinawa, Japan

**Keywords:** APP, TrkA, NGF, Alzheimer’s disease, CBFN, BiFC, proximity ligation assay

## Abstract

The amyloid precursor protein (APP) interacts with the tropomyosin receptor kinase A (TrkA) in normal rat, mouse, and human brain tissue but not in Alzheimer’s disease (AD) brain tissue. However, it has not been reported whether the two proteins interact directly, and if so, which domains are involved. Clarifying these points will increase our understanding of the role and regulation of the TrkA/APP interaction in normal brain functioning as well as in AD. Here we addressed these questions using bimolecular fluorescence complementation (BiFC) and the proximity ligation assay (PLA). We demonstrated that exogenously expressed APP and TrkA associate through their juxtamembrane/transmembrane domains, to form a complex that localizes mainly to the plasma membrane, endoplasmic reticulum (ER) and Golgi. Formation of the complex was inhibited by p75NTR, ShcC and Mint-2. Importantly, we demonstrated that the association between endogenous APP and TrkA in primary septal neurons were modified by NGF, or by drugs that either inhibit ER-to-Golgi transport or perturb microtubules and microfilaments. Interestingly, several agents that induce cell death [amyloid β (Aβ)-peptide, staurosporine and rapamycin], albeit via different mechanisms, all caused dissociation of APP/TrkA complexes and increased production of C-terminal fragment (β-CTF) APP fragment. These findings open new perspectives for investigating the interplay between these proteins during neurodegeneration and AD.

## Introduction

Alzheimer’s disease (AD) is a neurodegenerative disease that causes progressive decline of cognitive function in elderly people. The neuropathology of AD is marked by formation of extracellular amyloid-beta (Aβ) peptide plaques and intracellular neurofibrillary tangles (NFT), along with synapse and cell loss in selected brain areas including the cholinergic basal forebrain neurons (CBFN) (Zaboroszky et al., 2012). Impaired cholinergic neurotransmission has been proposed as potentially causal for the formation of plaques and tangles; these, in turn, exacerbate the cholinergic deficit (Kar et al., 2004; Adalbert et al., 2007; Belarbi et al., 2009; Vana et al., 2011). The viability of CBFN depends on nerve growth factor (NGF), which is produced by CBFN targets. Once bound and internalized, NGF is transported in a retrograde fashion to the cell body through interaction with the high-affinity TrkA and the low-affinity (p75NTR) neurotrophin receptors. Accumulating evidence highlights connections between the trafficking and signaling of NGF receptors with the processing and signaling of APP. Thus, perturbations in the expression or retrograde transport of NGF, TrkA or p75NTR can underlie CBFN dysfunction and altered APP and tau processing in AD (Capsoni et al., 2002; Isacson et al., 2002).

p75NTR, which is highly expressed in adult CBFN neurons, induces apoptosis in the absence of NGF (Casaccia-Bonnefil et al., 1996; Frade et al., 1996). The receptor also binds directly to Aβ-peptide, thereby enhancing peptide toxicity (Rabizadeh et al., 1994; Yaar et al., 1997). Moreover, p75NTR binds directly to APP. This interaction, which is inhibited by NGF and by Aβ-peptide, results in altered APP processing and reduced APP-mediated transcriptional activity (Fombonne et al., 2009). Interplay between TrkA and APP has also been reported. Decreased binding of NGF to TrkA leads to increased production of Aβ-peptide and to apoptotic death (Matrone et al., 2011; Triaca et al., 2016). TrkA interacts with APP (Matrone et al., 2011; Zhang et al., 2014). This depends on APP phosphorylation at Y682 (Tarr et al., 2002a; Matrone et al., 2011) and has been correlated with APP-processing (Tarr et al., 2002a; Barbagallo et al., 2010; Zhang et al., 2014), APP-mediated transcriptional activity, and cell death (Zhang et al., 2014). In turn, APP regulates the NGF/TrkA signaling pathway (Matrone et al., 2011), the sub-cellular distribution of TrkA (Matrone et al., 2011; Zhang et al., 2014), the endocytosis of the TrkA/p75NTR/NGF complex (Zhang et al., 2013), and the sensitivity of neurons to the trophic action of NGF (Matrone et al., 2011).

In consideration of these phenomena, NGF therapy has been proposed as an approach to counteract CBFN degeneration in AD patients (Williams et al., 2006).

Amyloid precursor protein and p75NTR interact directly (Zhang et al., 2013), but it is not known whether the same is true for APP and TrkA. In the present work, we used co-immunoprecipitation of selected TrkA and APP deletion mutants in transfected HEK293 cells to map interacting domains. Next, we used BiFC and PLA assays, two techniques that can identify interacting proteins, i.e., spaced within 7 and 15nm, respectively. BiFC is based on assembling a functional yellow fluorescent protein (YFP), by expressing two halves of YFP as fusions with cellular proteins that themselves interact. The interaction can then be detected by fluorescent microscopy and quantified by flow cytometry (FC) (Morell et al., 2008). *In situ* PLA uses antibody-coupled oligonucleotides together with DNA ligation and polymerization as a means to detect interaction between proteins of interest (Greenwood et al., 2015). PLA visualizes and quantifies contacts between specific proteins, in their normal context and at physiological expression levels, with extreme specificity. Moreover, PLA allows detection of single event with subcellular resolution, and generation of cell-to-cell statistics.

Using these approaches, we found that for APP and TrkA association their juxtamembrane and TMD domains respectively are sufficient. This interaction does not require tyrosine-phosphorylation of TrkA and is inhibited by p75NTR, ShcC and Mint-2. APP/TrkA complexes localize to the ER, Golgi, and plasma membrane. Formation of APP/TrkA complexes is promoted by NGF and is conversely reduced by agents that disrupt intracellular protein traffic, Aβ-peptide, and inducers of cell death. Interestingly, the loss of APP/TrkA complexes occurs rapidly, preceding the loss of cell viability.

## Materials and Methods

Rodent NGF was from Xiamen, Bioway.

### Antibodies

Antibodies used in this study were: mouse APP 22C11 (MAB348, Millipore), mouse APP-CT (clone C1/6.1 802801, Biolegend), rabbit APP-CT (A8717, Sigma Aldrich), rabbit APP-NT (A8967 Sigma Aldrich), rabbit monoclonal TrkA [EP1058Y] (Abcam, ab76291), mouse and rabbit pan-Trk (sc-7268 and sc-11, respectively), rabbit TrkA NT H-190 (sc-14024), rabbit TrkB (794) (Sc-12) and TrkC (Sc-117), goat polyclonal Calnexin (Sc-6465) and GM130 (Sc-16270), and mouse Bcl2 (sc-7382) were from Santa Cruz Biotechnology, Santa Cruz, CA, USA. Goat Anti-Choline Acetyltransferase (ChAT) antibody (AB144P) was from Millipore. Rabbit cleaved/caspase-3 (Asp175) (#9661) was from Cell Signaling

### Plasmid Vectors

pAPP-695 (1–695); pAPP-Δ-CT (1–651); pAPP-s-α (1–612), pAPP-C99 (597–695), and pAPP Sw/Ind (Swedish and Indiana mutation) plasmids were gently provided by Dr. Dennis Selkoe (Addgene: plasmids: #30137; 30143; #30147; #30146; #30145). pAPP-Y682G plasmid harboring mutation on the tyrosine 682 residue to glycine was obtained by site directed mutagenesis (Q5^®^ Site-Directed Mutagenesis Kit, New England Biolabs, E0554S). pAPP-C83 (613-695) plasmid was obtained by polymerase chain reaction (PCR) using pAPP-695 as PCR template.

pTrkA, pTrkA-Δ-CT, and pTrkA-Δ-NT plasmids were constructed starting from the DM38 vector coding for the human TrkA cDNA generously provided by Dr. Mariano Barbcid. Briefly, the Eco-RI fragment of TrkA cDNA was excited from DM38 vector, cloned in Eco-RI site of pBlue-script KS+ vector and then in pCDNA-3 vector as Hind-III-Xba-I fragment, to obtain pTrkA vector (1–799) coding for TrkA full length. pTrkA-ΔN-CT (1–488) vector was obtained inserting a stop codon into BspHI restriction site (1403 nt) of TrkA cDNA. pTrkA-Δ-NT (354–799) vector was obtained cloning the HindII-EcoRI TrkA fragment in frame and downstream the Ig leader sequence in pCDNA-3 vector. pCMV5-TrkA (K538A) was kindly provided by Dr. Moses Chao. TrkA-YFP plasmid was gently provided by Dr. Simon Alegoua and Dr. Annette Dolphin. APP-CFP plasmid was gently provided by Dr. Carmela Abraham. pCMV5-p75NTR plasmid was gently provided by Dr. Corinna Giorgi.

pTrkA-VN, pTrkA-VC, pErbB3-VN were described previously (Shen and Maruyama, 2011). For generation of pAPP-695-VN (APP-VN), pAPP-Sw/Ind-VN and pAPP-Y682G-VN vectors, the SacI-EcoRI fragment containing the FLAG tag sequence was removed from the vector pBiFC-VN173, to prevent the tag fusions with APP, and substituted with a new SacI-EcoRI fragment [lacking Flag, HindIII, NotI and EagI DNA sequences and formed by annealed complementary pair of oligonucleotides designed to harbor SacI and EcoRI sites], to obtain the pBiFC-D-Flag-VN vector.

*Primer forward*: 5′-cgtttagtgaaccgtcagaattgtctg-3′ and

*Primer reverse*: 5′-aattcagacaattctgacggttcactaaacgagct -3′

PCR products encoding human APP-695, APP-Sw/Ind and APP-Y682G proteins were amplified using pAPP-695, pAPP-Sw/Ind and pAPP-Y682G plasmids as PCR template using the following primer pairs:

*Primer forward*: 5′-gatatcatgctgcccggtttggcactgctcctgctg-3′ containing the EcoRV restriction and Kozak sequences

*Primer reverse*: 5′-gctctagacgtagcaaccggcgggtccctagccctcggaaccgtggagttctgcatctgctcaaagaactt-3′, containing sequences coding for linker STVPRARDPPVAT and Xba-I site for cloning APP in frame with VN in pBiFC-Δ-Flag-VN vector.

APP-C99-VN and APP-C83-VN vectors were generated after cloning C99 and C83 downstream and in frame with the signal peptide of APP in pCDNA3. From this vector both fused fragments were excised as EcoRI and XbaI fragment and cloned in pBiFC-VN vector. APP-Δ-CT-VN and APP-s-α-VN vectors were amplified by PCR and cloned in pBiFC-VN.

To generate TrkA-Δ-CT-VC vector, the fragment (residues 1–488) was amplified and cloned into EcoRI-Xho sites of BiFC vector. To generate TrkA-Δ-CT-VC vector, the fragment 354–799 was amplified and cloned downstream and in frame with the signal peptide of TrkA (residues 1–36) in pCDNA-3. The EcoRI-XhoI fragment was then excised and cloned in pBiFC-VC vector. To generate TrkA 354-488-VC vector, the TrkA sequence between residues 354 and 488, comprising the transmembrane domain, was cloned downstream and in frame with the signal peptide of TrkA in pCDNA-3, excised as EcoRI-XhoI fragment and cloned in BiFC-VC vector. The validity of the constructs were confirmed by DNA sequencing and WB analysis. Mint-2 plasmid was gently provided by Dr. Thomas C. Südhof. Plasmid for ShcC was from Origene (SC114141). DsRed-Rab-5 and DsRed-Rab11 plasmids were gently provided by Dr. Pagano (Addgene plasmids #13050, #12679 respectively). tdTomato-MannII-N-10 and mCherry-ER-3 plasmids were gently provided by Dr. Davidson (Addgene plasmids: #58110 and #55041 respectively).

### Cell Cultures

Septal neurons were prepared from embryonic day 17/18 (E17/18) pregnant Wistar rats (Charles River) as previously described (Triaca et al., 2016). All experiments regarding the establishment of primary neuronal cultures from rat brain were performed according to the national and international laws for laboratory animal welfare and experimentation. The experiments were performed according to a protocol communicated to the Italian Ministry of Health on February 18 th 2014, valid until February 18th 2017, in accordance with the guidelines and regulations of the Italian Law (DLGs n.116, 27/1/1992). HEK293 cell line (ATCC) were cultured in DMEM (Gibco) medium supplemented with 10% FBS (Invitrogen), penicillin/streptomycin in 5% CO_2_ humidified atmosphere, at 37°C and transfected with Lipofectamine 2000 (Life Technologies, according to the manufacturer’s instructions).

### Immunoprecipitation and Western Blot Analysis

Cells were lysed in RIPA buffer (10 mM Tris-HCl pH 7.6, 100 mM NaCl, 10 mM EDTA, 0.5% Nonidet P40, 0.5% sodium deoxycholate) in the presence of protease and phosphatase inhibitor cocktail (Sigma, Aldrich). Solubilized proteins were obtained in the supernatant after centrifugation at 13,000 rpm for 5 min. Protein concentrations were estimated by the Bradford procedure (Bio-Rad) for all samples except those containing SDS where the DC assay (Bio-Rad) was used. Co-immunoprecipitations were performed on pre-cleared solubilized proteins or supernatants using APP (22C11) antibody, pan-Trk antibody (B3) and non-relevant monoclonal mouse IgG antibody as control. WB were performed using rabbit APP-CT (1:2000), APP-NT (1:2000), rabbit monoclonal TrkA-CT (1:2000) and rabbit TrkA-NT (1:1000) antibodies. Densitometry of the WB was analyzed with the Image J software (NIH) and normalized to corresponding reference protein β-actin.

### Immunofluorescence

Primary septal cell cultures were fixed for 20 min in phosphate-buffered saline (PBS) containing 4% paraformaldehyde (PFA) and permeabilized for 5 min with 0.2% Triton X-100 at room temperature (RT). Then, they were incubated in 0.1 M ammonium chloride for 30 min at RT to reduce autofluorescence and blocked with 10% normal donkey serum (NDS, Jackson Immunoresearch) in PBS for 60 min at RT. Primary antibody incubations were performed overnight at 4°C. Cells were then washed three times with PBS plus 0.1% Tween 20 at RT, incubated with Alexa-labeled secondary antibodies (Invitrogen, anti-mouse-546, anti-rabbit-488) for 1 h at RT in the same buffer, counterstained with 4′,6-diamidino-2-phenylindole (DAPI) and mounted. Images were visualized with a confocal microscope (TCS SP5, Leica Microsystem GmbH Wetzlar, Germany) using a 60× 1.35 NA oil immersion objective and acquired with the LEICA Application Suite software (Advanced Fluorescence Lite, Leica).

For the quantitative analysis, all the images were acquired with identical settings of brightness and contrast. The APP and TrkA immunoreactivities were measured through densitometric analysis by using the ImageJ software (version 1.41^1^). Images were converted to eight-bit images containing grayscale values from 0 (black) to 255 (white). After background subtraction, the APP and TrkA cell-associated signals were quantified by manually outlining individual cells. The integrated value was calculated by summing the gray values of each pixel, and the mean intensity value was calculated by dividing the integrated intensity by the number of pixels.

### Förster Resonance Energy Transfer (FRET)

For standard acceptor photobleaching FRET microscopy, HEK293 cells were seeded on coated glass coverslips and co-transfected with a vector encoding APP-CFP and a vector encoding TrkA-YFP. Twenty-four hours after transfection, cells were fixed with 4% paraformaldehyde for 10 min and, following three washes, were placed on slides and embedded in mounting medium. FRET studies were performed on a confocal microscope (TCS SP5, Leica) using the implemented FRET acceptor photobleaching wizard. Acquisition settings were as follows: objective Plan-Apochromat × 63/1.4 NA oil immersion, pinhole 2 Airy units, image size 512 × 512. Prebleach and postbleach images were serially recorded of APP-CFP [(exicitation at 458 nm and emission at 475/500 nm (donor channel)] and Trk-YFP [exicitation at 515 nm and emission at 530/630 (acceptor channel)]. Low laser intensities were used to avoid bleaching effects during acquisition, selection of the cells were made by visualizing only the donor channel to prevent premature partial bleaching of the acceptor. The acceptor was bleached with high intensity (100%) power at the 543 nm laser line for 10 iterations. This iteration time was found to be effective for bleaching TrkA-YFP in pilot experiments. The change in the fluorescence intensity between pre- and postbleach donor values (efficiency, ***E***) was calculated using the formula ***E*** = (donor after–donor before) × 100/donor after, and was shown as a percentage. We analyzed at least10 cells from each of three independent experiments.

### Bimolecular Fluorescence Complementation (BiFC)

Formation of complexes containing APP/TrkA ectopically expressed in transfected HEK293 cells was measured by BiFC (Morell et al., 2008). For confocal microscope analysis, 50,000 HEK293 cells per well were seeded on coverslips coated with poly-L-Lysine (50 μg/ml, Sigma) and cultivated in 5% CO2 at 37°C. On the next day, cells were co-transfected with 400 ng of the expression vectors (200 ng each) indicated in each experiment using Lipofectamine. The same amount of DNA was used for each co-transfection. To evaluate the role of p75NTR, ShcC and Mint-2 in APP/TrkA interaction, HEK293 cells, plated on 12-well plates were transfected with TrkA-VC and APP-VN and expression vector coding for un-tagged p75NTR, ShcC and Mint-2 at ratio of 1:1:3 respectively (200: 200: 600 ng of each plasmid). For mock, single and double plasmid transfection, pcDNA-3 empty vector was added to standardize the plasmid amount. 18–24 h after transfection, cells were washed twice with PBS stained with DAPI and analyzed at confocal microscope.

### Flow Cytometry (FC)

For FC analysis, 100,000 HEK293 cells per well were seeded in 12-well plates and cultivated in 5% CO_2_ at 37°C. On the next day, transfections with equal vector amounts (1 μg of total amount of DNA transfected) were performed. Eighteen to twenty-four hours after transfection, cells were washed with PBS, trypsinized, pelleted and suspended in 1 ml PBS. The flow cytometric analyses of the single-cell suspensions were performed using a fluorescence-activated cell sorter (FACS; FACScan; BD Biosciences, San Jose, CA, USA) equipped with an argon laser emitting at 488 nm. Analysis was restricted to live cells by gating cells that exhibited forward and side scatter features typical of live cells. Data from the acquisition of 20,000 cells were analyzed using CellQuest software (BD Biosciences). Among the subsequent Histogram statistics the “average channel number or linear value of events within a marker” meaning the Mean statistic value, was chosen for the following analysis.

### *In situ* Proximity Ligation Assay (PLA)

The interaction between APP and TrkA by PLA assay (Fredriksson et al., 2002) was detected using the corresponding two primary antibodies raised in different species. Species-specific secondary antibodies (PLA probes), each with a unique short DNA strand (MINUS and PLUS) attached to it, bind to the primary antibodies. When the PLA probes are in close proximity the DNA strands can interact through a subsequent addition of two other circle-forming DNA oligonucleotides. After the amplification reaction of the DNA circle a fluorescent signal is generated by labeled complementary oligonucleotide probes. To perform PLA assay, primary septal cultures at 10–12 days *in vitro* (DIV), treated as indicated, were immediately fixed in 4% PFA for 15 min and thereafter subject to *in situ* PLA using Duolink In Situ Detection Reagents Red kit (Sigma Aldrich, DUO92008) according to the manufacture’s instruction. All incubations were performed in a humidity chamber. Briefly, coverslips were blocked with NDS for 60 min at RT and then incubated with different anti-APP and anti-TrkA antibodies, diluted in antibody diluent (DUO82008), overnight at 4°C. Coverslips were washed three times for 5 min PBS 1X and then incubated with PLA Probe Anti-mouse MINUS (DUO92004) and Anti-rabbit PLUS (DUO92002) for 1 h at 37°C. Then, coverslips were washed three times for 5 min in 1X washing buffer A (DUO82049) buffer under gentle shaking and then incubated with a DNA ligase diluted in Ligation buffer for 30 min at 37°C. Coverslips were washed three times for 2 min in 1X washing buffer A under gentle shaking and incubated with DNA polymerase diluted in Amplification buffer for 90 min at 37°C. Finally, coverslips were rinsed for 10 min in Duolink 1X washing buffer B (DUO82049) and for 2 min in washing buffer B 0.01X. Dried coverslips were mounted with mounting medium (DUO82040) to stain the nuclei. At the end of this procedure, each TrkA/APP complex generated a fluorescent red spot.

### Microscope/Images Acquisition

Fluorescence images were acquired on a TCS-SP5 confocal laser scanning microscope (Leica Mycrosystem GmbH Wetzlar, Germany) using 63 × 1.35 NA oil immersion objective. High resolution images were acquired as z-stack with a 0.5 μm z-interval (at least 10 planes), and converted to maximum projection images (to avoid subjectivity in the choice of the plane to be analyzed) with the LASAF software platform (Leica Microsystem) in the TIFF format.

### Quantification of PLA Signal

Quantification of PLA signal was performed from at least 20 cells from different experiments as previously reported Trifilieff et al. (2011). High-resolution (63 × 1.35 NA) were analyzed in ImageJ (NIH) to calculate the number of PLA puncta. Image were first smoothed and threshold was selected manually to discriminate PLA puncta from background fluorescence. Once selected, this threshold was applied uniformly to all images in the sample set. The built in macro “Analyze particle” was then used to count all particles in the thresholded image. Objects larger than 5 μm^2^ were rejected, thereby effectively removing nuclei. The remaining objects were counted as PLA puncta. Interactions were quantified by counting the number of dots per cell. In the different figures, each bar (Mean ± SEM) represents the mean obtained from the quantification of signals observed in 20–30 cells chosen randomly in five different fields from 3 independent experiments.

### Induction of Cell Death

For Aβ toxicity, primary septal cultures (10 DIV) were exposed to 20 μm of Aβ1-40 (Abcam, Cambridge, UK). Aβ-peptide stock solutions at a concentration of 1 mg/ml were prepared in PBS (0.01 M NaH2PO4, 0.15 M NaCl, pH 7.4) and stored to -20°C. Macroautophagy was induced with rapamycin (10 nM) a suppressor of mTOR kinase. To induce apoptosis, neurons (10 DIV) were exposed to the protein kinase inhibitor staurosporine (Sigma Aldrich) (30 nM). Controls were exposed to the vehicle.

### Assessment of Cell Viability

Viability was quantified by the 4,5-dimethylthiazol-2-yl)-2,5-diphenyltetrazolium bromide (MTT) assay (Esposito et al., 2012) and by counting the number of intact nuclei in a haemacytometer, after lysing the cells in detergent-containing solution (Soto and Sonnenschein, 1985; Volonte‘ et al., 1994; Esposito et al., 2012). This latter method has been shown to be reproducible and accurate and to correlate well with other methods of assessing cell survival-death (Stefanis et al., 1997; Stefanis et al., 1999). Briefly, the culture medium was removed and replaced with 0.5 ml of a detergent containing lysing solution (0.5% ethyl- hexadecyldimethylammonium bromide, 0.28% acetic acid, 0.5% Triton X-100, 3 mM NaCl, 2 mM MgCl2 in PBS pH 7.4 diluted 1/10). After 2 min, the suspension of cells was collected. Nuclei from originally viable cells were then quantified by counting in hemocytometer as the detergent-containing solution selectively dissolves nuclei of dying cells. Nuclei from viable cells thus appear as phase-bright intact circles surrounded by a dark ring. Broken or damaged nuclei were not included in the count.

### Statistical Analysis

Values are mean ± SEM (standard error of mean) or ±SD (standard deviation) as indicated. Statistical analysis was carried out using GraphPad Prism 6 software (San Diego, CA, USA). For multiple testing ANOVA followed by *post hoc* testing (Bonferroni’s Multiple Comparison Test) was used. A *p*-value of less than 0.05 was considered statistically significant (^∗^*p* < 0.05; ^∗∗^*p* < 0.01; ^∗∗∗^*p* < 0.001).

## Results

### Domains Involved in TrkA/APP Interaction

**FIGURE 1:**
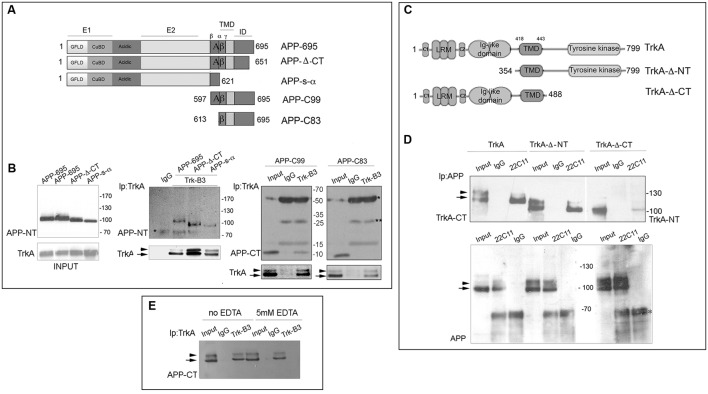
**Identification of the APP and TrkA regions involved in APP/TrkA interaction.** **(A)** Schematic diagram of APP constructs used in this study. All constructs are derived from the human APP-695 isoform. This transmembrane glycoprotein consists of 695 amino acids and can be divided into an extracellular region, a transmembrane domain (TMD) and a small intracellular domain (ID) the extracellular regions is divided into the E1 and E2 domains, linked by an acidic domain. The domain E1 consists of a growth factor-like domain (GFLD) and the following copper-binding domain (CuBD). **(B)** Cell lysates of HEK293 cells, transfected with the indicated plasmids, were immunoprecipitated with mab Trk (B3) or mab Bcl2 (C-2) (IgG) as control and probed with rabbit anti C-terminal (CT), N-terminal (NT) APP antibodies and rabbit TrkA-CT (Abcam ab76291). Total protein extract (30 μg unbound) (INPUT) was also loaded as positive internal control of electrophoretic mobility and immunoprecipitation efficiency. Each co-transfection has been performed at least three times with similar results. **(C)** Schematic diagram of TrkA constructs used in this study. All constructs are derived from human TrkA. This receptor consists of 796 amino acids and can be divided in the extracellular ligand-binding domain, [comprising leucine-rich motifs (LRR), two cysteine clusters (C1 and C2) and two immunoglobulin (Ig)-like domains], the TMD, and the intracellular tyrosine kinase domain. **(D)** Cell lysates of HEK293 cells transfected with APP-695 and TrkA constructs were immunoprecipitated with mab 22C11 or mab Bcl2 (C-2) (IgG) as control and then probed with rabbit anti-TrkA-CT (Abcam, ab76291) and TrkA-NT (H-190 (sc-14024). INPUT corresponds to 30 μg of total unbound extract as in **(B)** ^∗^ and ^∗∗^ indicates the IgG heavy and light chains respectively. **(E)** Immunoprecipitation with anti-TrkB3 from lysate of HEK293 cells, transfected with APP-695 and TrkA constructs, in the presence or absence of 5 mM EDTA. INPUT corresponds to 30 μg of total unbound extract as in **(B)**. Arrow and arrowhead indicate the partially glycosylated and fully glycosylated forms of TrkA, and the immature and mature forms of full length APP.

To identify APP region(s) that associates with TrkA, HEK293 cells were co-transfected with a TrkA full-length construct plus a series of APP full-length and deletion constructs (**Figure 1A**). Cell lysates immunoprecipitated with an anti-TrkA antibody were analyzed by Western blot (WB) with rabbit antibodies against N-terminal (NT) or C-terminal (CT) of APP. We found that the APP juxtamembrane region comprised between β and α secretase cleavage sites was necessary for co-immunoprecipitation with TrkA as shown by co-immunoprecipitation of APP-C99 and TrkA and lack of co-immunoprecipitation of APP-C83 and TrkA (**Figure 1B**). Similarly, by co-immunoprecipitation of APP with TrkA deletion mutants (**Figure 1C**), we demonstrated that APP interacts with a TrkA fragment which lacks 353 NT amino acids and includes the intracellular and transmembrane domains (TrkA-Δ-NT). Moreover, APP still interacts, albeit with reduced efficiency, with TrkA-ΔCT lacking the intracellular CT 311 amino acids but including the extracellular and transmembrane domains (**Figure 1D**). Taken together, these data suggests that the transmembrane, the only one shared by TrkA-Δ-NT and TrkA-Δ-CT, is important for interaction with APP and that the intracellular (IC) domain of TrkA may contribute to the binding to APP since its deletion decreases the amount of co-immunoprecipitated APP. Moreover, the level of immunoprecipitated proteins was similar in the absence and in the presence of EDTA. This suggests that the formation of TrkA APP complex does not require divalent cations, such as cupper and zinc, known to influence APP oligomerization (**Figure 1E**).

### FRET and BiFC Analysis of APP/TrkA Interaction

**FIGURE 2:**
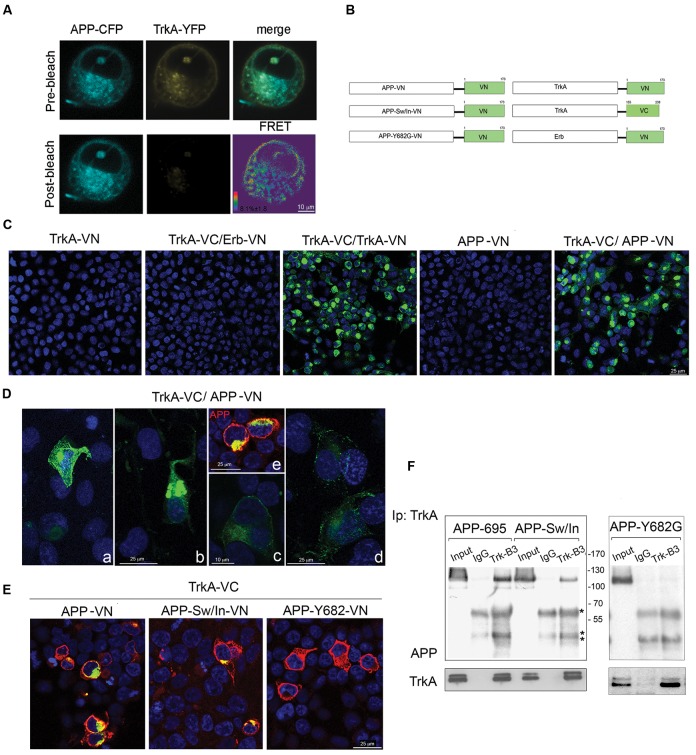
**Visualization of TrkA-APP protein interaction using FRET and BiFC analysis.** **(A)** Confocal microscope images of FRET acceptor photobleaching assay of HEK293 cells co-transfected with APP-CFP and TrkA-YFP. The CFP and YFP images before and after YFP photobleaching, the merged image of CFP and YFP prior to photobleaching, and pseudo-colored image showing an FRET efficiency values map. **(B)** The schematic diagram of dimerization pairs used in the BiFC analysis. **(C)** HEK293 cells were transfected with TrkA-VN alone, TrkA-VC and ERB-VN, APP-VN alone, TrkA-VC and TrkA-VN, TrkA-VC and APP-VN. Fluorescence images of HEK293 cells expressing the proteins indicated in each panel were acquired by confocal microscopy 18–24 h after transfection. **(D)** Four images of cells transfected with TrkA-VC/APP-VN were selected to show cells with different level of protein expression (a, b: high level of expression; c, d: low level of expression) but similar pattern of subcellular distribution; e, APP staining with mab 22C11 (red). **(E)** BiFC analysis shows that TrkA-VC interacts also with APP-Sw/In-VN but not with APP-Y682G-VN, despite this latter is efficiently expressed when transfected (see immunostaining for APP performed with mab 22C11, red). **(F)** Cell lysates of HEK293 co-transfected with TrkA and APP-Sw/In or APP-Y693G were immunoprecipitated with mab Trk (B3) antibody or mab Bcl2 (IgG) as control followed by immunoblot with rabbit APP-N-terminal and rabbit TrkA.^∗^ and ^∗∗^ indicate the IgG heavy and light chains respectively.

To investigate the physical association of APP and TrkA FRET was performed in HEK 293 cells transfected with TrkA-YFP and APP-CFP plasmids. The average FRET efficiency values measured was 8.1% ± 1.8 (**Figure 2A**). In a previous paper, the FRET behavior of two tandem CFP-YFP fusion proteins has been investigated (Karpova et al., 2003). A FRET efficiency value of 7.96% ± 0.38 was achieved for the CFP-YFP tandem proteins. Comparison of our APP-YFP value with that of the CFP-YFP tandem construct suggests that APP and TrKA are in close proximity in the interacting complex.

Formation of APP/TrkA complexes and their subcellular localization were further investigated by BiFC assay (Kerppola, 2006). We co-transfected HEK293 cells with an expression vector encoding the aminoterminal fragment of venus fluorescent protein (residues 1-173, VN) (Nagai et al., 2002) fused in frame to the C-terminus of APP-695, hereon named APP-VN, with vector encoding the C-terminus of TrkA fused in frame with carboxyl fragment of venus fluorescent protein (residues 155-238, VC), hereon named TrkA-VC. Negative and positive controls consisted of transfection of TrkA-VC with ErbB3-VN, ErbB3 being a receptor for ligands of EGF family with no known interaction with TrkA, and TrkA-VC with TrkA-VN known to homodimerize (Shen and Maruyama, 2011) respectively (**Figure 2B**). As reported in **Supplementary Figure S1**, APP tagged with VN is expressed with the expected size and processed normally.

As shown in **Figure 2C**, TrkA-VC and APP-VN reconstituted a functional fluorophore indicating that they are in close proximity. The strong BiFC signal indicates that TrkA/APP form a complex, with a half-life that is longer than 50 min, the time needed to produce a BiFC signal once the two halves bind to each other (Hu and Kerppola, 2003). This result suggests that the interaction did not result in the degradation of the TrkA/APP complex. As expected, no fluorescence was observed in cells expressing TrkA-VC/ErbB3-VN, TrkA-VN and APP-VN alone. TrkA/APP complexes were observed not only in the plasma membrane, but also inside the cells (**Figures 2D,E**). The level of intracellular complexes was much higher than that in the plasma membrane and exhibited distribution pattern within the cells suggestive of an ER, and Golgi localization (see **Supplementary Figure S5**).

To exclude that the observed interaction between APP and TrkA proteins was due to their over-expression, we selected cells expressing different levels of APP and TrkA (**Figure 2D**) and found that cells with barely detectable levels of BiFC signal (**Figure 2D**, c,d) exhibited a similar subcellular distribution pattern as those having a strong BiFC signals (**Figure 2D**, a,b).

Interestingly, we detected BiFC also when TrkA-VC was transfected with APP-VN bearing the Sw/In mutations (**Figure 2E**), suggesting that mutations K596N, M597L and V642F do not preclude APP binding to TrkA. Consistently, co-immunoprecipitation confirmed that TrkA interacts with APP-Sw/In (**Figure 2F**). By contrast, APP with the mutation Y682G in the ^682^YENPTY^687^ domain, which is instrumental for the association with TrkA (Matrone et al., 2011) was unable to form BiFC signal (**Figure 2E**) and to immunoprecipitate TrkA (**Figure 2F**). Of note, APP bound also the TrkA K538A mutant which is unable to autophosphorylate (**Supplementary Figure S2**).

### Quantification of BIFC Analysis

**FIGURE 3:**
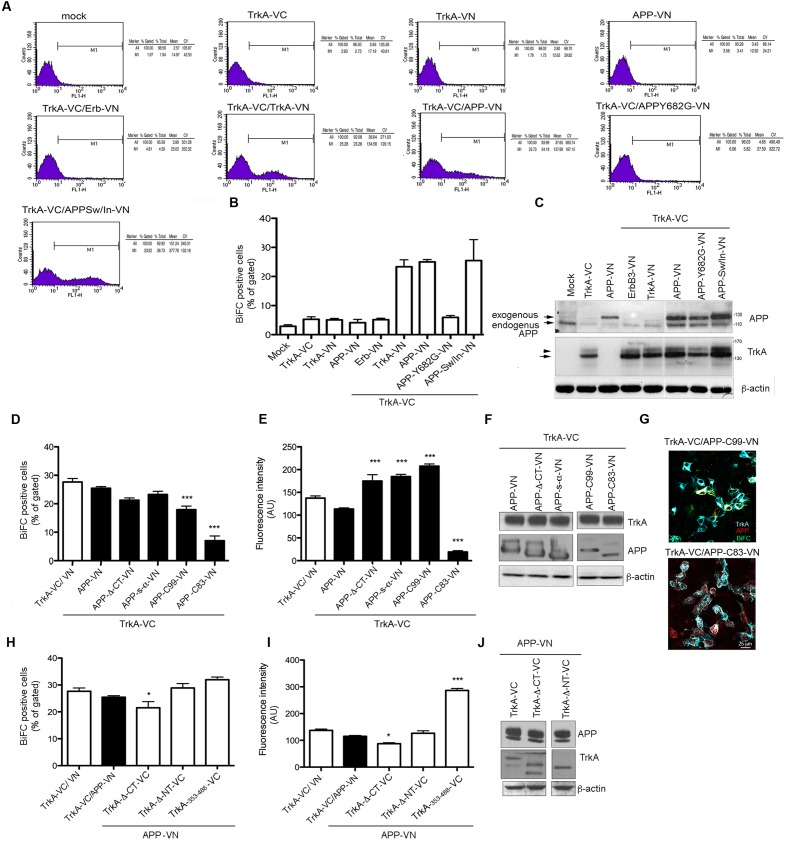
**Quantification of BiFC by FACS analysis.** **(A)** FACS analysis of HEK293 cells transfected with the indicated constructs. The M1 region was chosen to best estimate the percentage of cells exhibiting BiFC signal in co-transfection of TrkA-VC and APP-VN. The percentage gated and total, mean and coefficient of variation (CV) in each experiment are shown in the right panel. **(B)** The average of the percentage gated in (a) from three independent transfections. Error bars represent SEM of the average of percentage gated (*n* = 3). **(C)** Half of the cells transfected for FACS analysis were collected, lysed and analyzed by Western blotting with either rabbit anti-APP-NT and rabbit anti-TrkA-NT (H190) antibodies to ascertain equal transfection efficiency. Arrow and arrowhead indicate TrkA bands corresponding to partially glycosylated and fully glycosylated forms, respectively. **(D,E)** Quantification by FACS analysis of HEK293 cells transfected with the TrkA-VC plus the indicated APP mutant constructs, of the % BiFC positive cells and the mean fluorescence intensity (AU). Results are mean ± SEM of three independent transfections performed in triplicate. ^∗∗∗^ < 0.0001; vs. TrkA-VC/APP-V. **(F)** Western blot analysis with either rabbit anti-APP-NT and APP-CT and rabbit anti TrkA antibodies to ascertain equal transfection efficiency. **(G)** Visualization of BiFC signal (green) and TrkA (blue) and APP (red) immunostaining in HEK293 transfected with TrkA-VC/APP-C99-VN but not in cells transfected with TrkA-VC/APP-C83-VN. Fluorescence images were acquired by confocal microscopy 18-24 h after transfection. **(H,I)** Quantification by FACS analysis of HEK293 cells transfected with the APP-VN plus the indicated TrkA mutant constructs, of the % BiFC positive cell and the mean fluorescence intensity (AU). Results are mean ± SEM of three independent transfections performed in triplicate. ^∗^ < 0.01; ^∗∗∗^ < 0.0001; vs. TrkA-VC/APP-VN, One way Anova with Bonferroni *post hoc* test. **(J)** Western blot analysis either rabbit anti-TrkA-NT, TrkA-CT and rabbit anti APP antibodies to ascertain equal transfection efficiency.

BiFC fluorescence intensity was quantified by FACS analysis to determine the efficiency of fluorescence complementation in cells transfected with the various form of APP and TrkA. Mock transfected cells, as well as cells transfected with either TrkA-VN, APP-VN or TrkA-VC produced no fluorescence signal. Conversely, a peak of fluorescence was detected when APP-VN and TrkA-VC were co-transfected, with a mean value of 138.65 (arbitrary fluorescence units), similar to that of positive control TrkA-VC/TrkA-VN (134.58) (**Figure 3A**). The proportion of co-transfected cells showing BiFC signal was 25% ± 0.78 of BiFC positive cells for TrkA-VC/APP-VN and a 23.35% ± 2.38 of BiFC positive cells for TrkA-VC/TrkA-VN. These values were very close to that of control cell transfected with plasmid coding full length YFP fused in frame to C-terminus of APP-695 (APP-YFP) or C-terminus of TrkA (TrkA-YFP) (27% ± 4 and 25.5 ± 5.3 respectively), suggesting that the interaction occurs in all transfected cells.

In cells co-transfected with TrkA-VC/APP-Y682G-VN fluorescence intensity and % of BiFC positive cells were indistinguishable from that of negative control cells transfected with TrkA-VC/ErbB3-VN (**Figure 3B**). This finding is consistent with fluorescence microscopy analysis, which revealed the lack of BiFC signal for this mutant form of APP as well for the negative control TrkA-VC/ErbB3-VN (**Figure 2D**). Cells transfected with various constructs produced comparable amounts of protein (**Figure 3C**).

Co-transfection of full length TrkA with APP deleted constructs (**Figure 1A**) confirmed data obtained by immunoprecipitation. Indeed, BiFC signal was produced when TrkA-VC was co-transfected with APP-Δ-CT-VN, APP s-α-VN or APP C99-VN vectors (**Figures 3D,E,F**). Interestingly, no BiFC signal was evident for APP-C83-VN despite co-transfected cells express efficiently TrkA and C83 proteins as showed by WB and immunofluorescence analysis (**Figures 3F,G**). Co-transfection of N or CT deleted TrkA-VC fusion constructs with APP-VN produced BiFC signal. Moreover, we found that 354–488 TrkA fragment fused to VC was by itself capable of generating a strong BiFC when co-transfected with APP 695-VN (**Figures 3H,I,J**), suggesting that the transmembrane domain of TrkA was sufficient for interaction with APP.

### TrkA/APP Interaction Is Inhibited by p75NTR, ShcC and Mint-2

Many proteins can be brought in proximity to each other by a partner that functions as a scaffold for the assembly of a trimeric protein complexes. Simultaneous binding by two proteins in the vicinity of each other on the same scaffold results in increased BiFC signal (Riese et al., 2013; Mo et al., 2014). We therefore investigated whether the TrkA/APP interaction could be affected by the co-expression of p75NTR, ShcC and Mint-2, well-known TrkA and APP partners.

**FIGURE 4:**
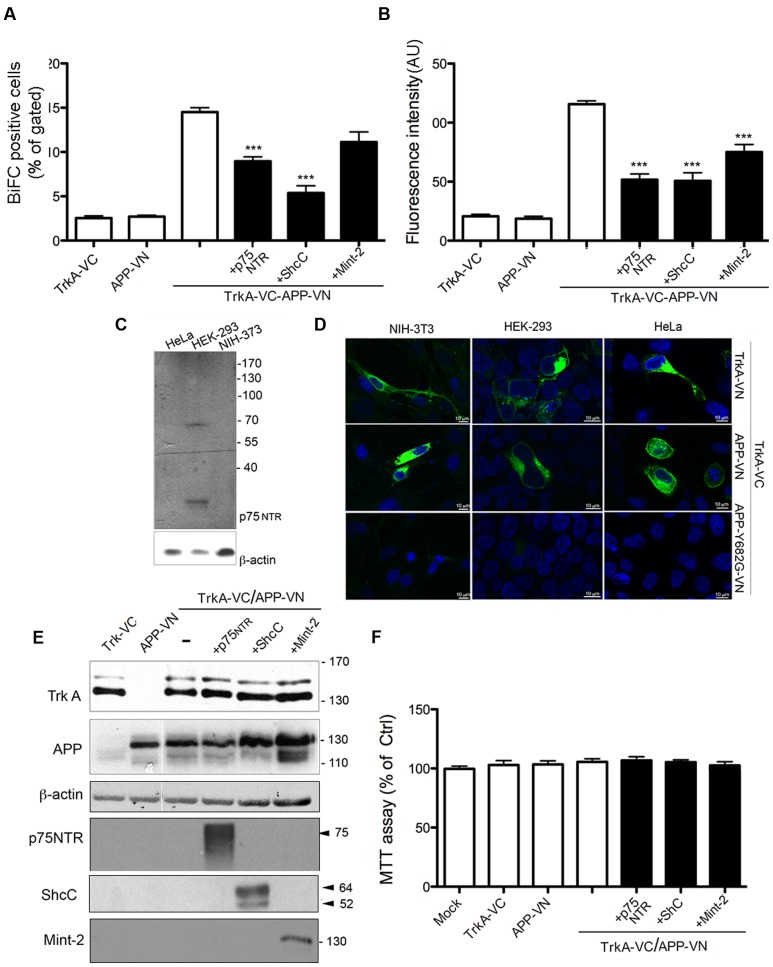
**TrkA/APP interaction is inhibited by low-affinity NGF receptor p75, ShcC and Mint-2.** **(A,B)** Quantification by FACS analysis of HEK293 cells transfected with the indicated constructs of the % BiFC positive cell and the mean fluorescence intensity. Results are mean ± SEM of three independent transfections performed in triplicate. ^∗∗∗^ < 0.0001; compared to TrkA-VC/APP-VN, One way Anova with Bonferroni *post hoc* test. **(C)** Western blot analysis showing expression of p75NTR in cell lines indicated. **(D)** Visualization of TrkA/APP interaction by BiFC analysis in HEK293, NIH-3T3 and HeLa transfected with the indicated plasmids. Fluorescence images were acquired by confocal microscopy 18–24 h after transfection. **(E)** Half of the cells transfected for FACS analysis were collected, lysed and analyzed by Western blotting with either rabbit anti APP-NT (A8967, Sigma Aldrich), rabbit anti TrkA-NT (H190), rabbit anti p75 NTR polyclonal antibody (N 3908, Sigma Aldrich), mouse anti ShcC, rabbit anti-Mint2 (sc-30135, X11β M-220 Santa Cruz Biotecnology). **(F)** Cell viability determined by the MTT assay 24 h after transfection. Results are the means (±SEM) of duplicate determinations from three independent experiments and are reported as percentage of mock transfected cells.

TrkA-VC and APP-VN were co-expressed with untagged p75NTR in HEK393, and BiFC analysis was performed 24 h later. There was no increase in BiFC efficiency, e.g., in the number of BiFC positive cells or in the fluorescence intensity of BiFC complex, the latter correlating with the size and stability of the complexes (Riese et al., 2013; Mo et al., 2014). Indeed, there were significant decreases in both of these parameters. First, we measured a significant decrease in the fraction of BiFC-positive cells, when compared with cells transfected solely with TrkA-VC and APP-VN (8.96 ± 0.48 and 14.50 ± 05 respectively; *p* = 0.0001) (**Figure 4A**; **Supplementary Figure S3**). Similarly, cells expressing p75NTR showed a significant decrease in mean fluorescence intensity (51.7196 ± 4.8 and 115 ± 2.67 respectively; *p* = 0.0001) (**Figure 4B**). These data suggest that, rather than promoting the interaction between APP and TrkA, p75NTR actually opposes it, likely by sequestering APP or TrkA (**Figure 4C**). Consistent with this observation, we found that APP-VN and TrkA-VC also form complexes in cells, such as NIH-3T3 and HeLa, which do not express p75NTR (**Figures 4C,D**).

We also evaluated the possible contribution of the neurospecific adapator protein ShcC in mediating TrkA/APP interaction. This adaptor protein was reported to contact the phosphotyrosine motif of TrkA and APP through its NT PTB domain (Dikic et al., 1995; Tarr et al., 2002b) and possibly the phosphotyrosine motif of APP through its C-terminal Src-homology2(SH2) domain (Tamayev et al., 2009). ShcC transfection, reduced the BiFC signal generated from co-expression of TrkA-VC/APP-VN of about 60% [(BiFC positive cells: 5.37 ± 0.8 and 14.50 ± 05 respectively; *p* = 0.0001; fluorescence intensity: 50.70 ± 11.94 and 115 ± 2.67; *p* = 0.0001)] (**Figures 4A,B**; **Supplementary Figure S3**) supporting that ShcC, like p75NTR, prevents instead of promoting TrkA and APP interaction.

Finally, a similar effect of Mint-2 in antagonizing TrkA/APP interaction was observed. This neural adaptor protein, involved in synaptic vesicle docking and exocytosis, is able to contact TrkA (Zhang et al., 2009) as well as APP (Biederer et al., 2002). Its expression resulted in a decrease by ∼30% of fluorescence intensity compared to control cells (73 ± 8.1 and 115 ± 2.67 respectively, *p* = 0.01), with a slight decrease in the number of BiFC positive cells (11.3 ± 1 and 14.50 ± 05 respectively) (**Figures 4A,B**; **Supplementary Figure S3**). The lack of involvement of ShcC and Mint-2 in promoting TrkA/APP complex formation is in accord with the co-immunoprecipitation of TrkA with APP-Δ-CT which lacks the motif ΔY^682^ENPTY^687^ necessary for interaction with the PTB motif of ShcC and Mint-2 (**Figure 1B**).

Notably, the extent of TrkA-VC and APP-VN expression was not decreased by co-transfection of p75NTR, ShcC or Mint-2, as shown by WB analysis (**Figure 4E**), indicating that the reduced BiFC signal is not due to a variations in expression level of proteins in the different conditions. Moreover, cell viability was not affected in any of the transfection indicating no overt cytotoxicity of the ectopically expressed proteins (**Figure 4F**).

Although our data demonstrated that neither p75NTR nor ShcC nor Mint-2 is required for promoting association between APP and TrkA, other factors such as sortilin (Vaegter et al., 2011; Gustafsen et al., 2013) might be involved. Alternatively, APP and TrkA might interact directly.

### Visualization of TrkA/APP Complex *In situ* by Proximity Ligation Assay in Septal Primary Neurons

**FIGURE 5:**
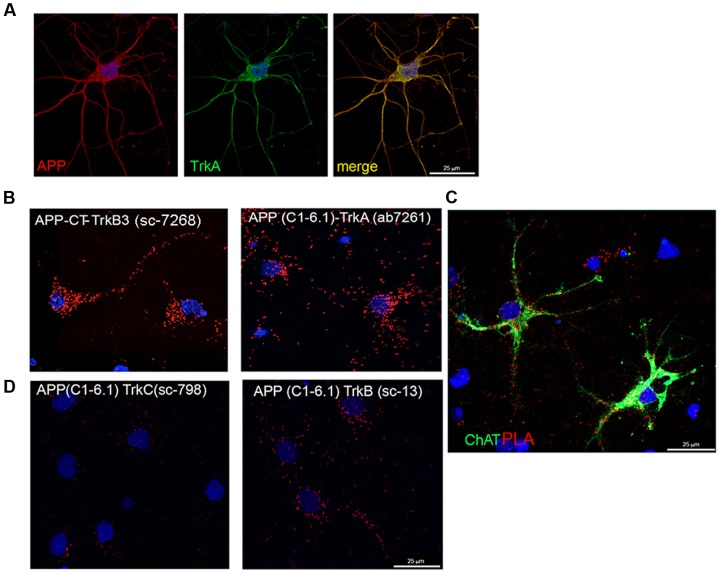
**TrkA and APP interaction in septal primary neurons measured by proximity ligation assay.** **(A)** Confocal microscopy analysis of double-staining of APP (rabbit APP-CT A8717, red) and Trk (mouse Trk B3, green) in primary septal neurons showing co-localization of APP and TrkA. **(B)** The TrkA/APP complex was visualized by PLA, which generates red dots when the two proteins are in close proximity. Mouse and rabbit anti TrkA-CT (mouse Trk B3 and rabbit TrkA ab7261) and anti APP-CT (rabbit APP-CT A8717 and mouse APP clone C1-6.1) were used as primary antibodies and anti-mouse MINUS and anti-rabbit PLUS were used as secondary antibodies. Red fluorescent dot represents single interaction between TrkA and APP. **(C)** In addition to PLA (red dots), rat primary septal neurons were immunostained with goat anti ChAt (green) and with DAPI for nuclei (blue). **(D)** PLA assay using mouse anti APP (C1-6.1) and rabbit anti TrkB (sc-119) or TrkC (sc-117).

We next used PLA assay to detect interaction between endogenous TrkA and APP in primary cultures of rat septal neurons. Preliminary confocal fluorescence microscopy analysis confirmed a strong colocalization of the signal for the two proteins (**Figure 5A**; Triaca et al., 2016). Next, we used a panel of anti TrkA and anti APP antibodies in PLA assay.

A strong signal in a form of spots reflecting TrkA-APP interaction, was detected with different specific antibodies against CT of TrkA and APP (**Figure 5B**). No or faint signal was observed with antibodies against the NT of TrkA and APP despite these antibodies are useful for staining APP and TrkA in immunofluorescence experiments (**Supplementary Figure S4B**). This finding suggests that TrkA and APP are spatially oriented in a manner that the distance between their NTs is greater than the one required generating a PLA signal. As control, no signal was detected by omission of one of the primary antibodies or use of an unrelated antibody (**Supplementary Figure S4A**).

Strong PLA signals were observed throughout the entire neuron, from the cell body to the distal parts of the neurites (**Figure 5B**), the majority of PLA signal being restricted, as expected, to cholinergic (choline acetyltransferase, Chat positive) neurons (**Figure 5C**). Trk B3 antibody is a pan Trk antibody which does not distinguish between TrkA, TrkB and TrkC receptors. To assess the contribution of hypothetical TrkB/APP and TrkC/APP complexes to the signal observed by PLA, we repeated the assay with rabbit anti-TrkB-specific (Sc-13) or anti-TrkC-specific (Sc-798) and mouse anti-APP-CT (clone C1/6.1) antibodies. Few scattered PLA dots were detected in both conditions (**Figure 5D**) suggesting that APP might form complex also with TrkB and TrkC, which are both expressed, although to a lower level than TrkA, in cholinergic neurons (Salehi et al., 1996; Mufson et al., 2002).

### TrkA/APP Complex Is Modulated by NGF and Cellular Trafficking Perturbations

**FIGURE 6:**
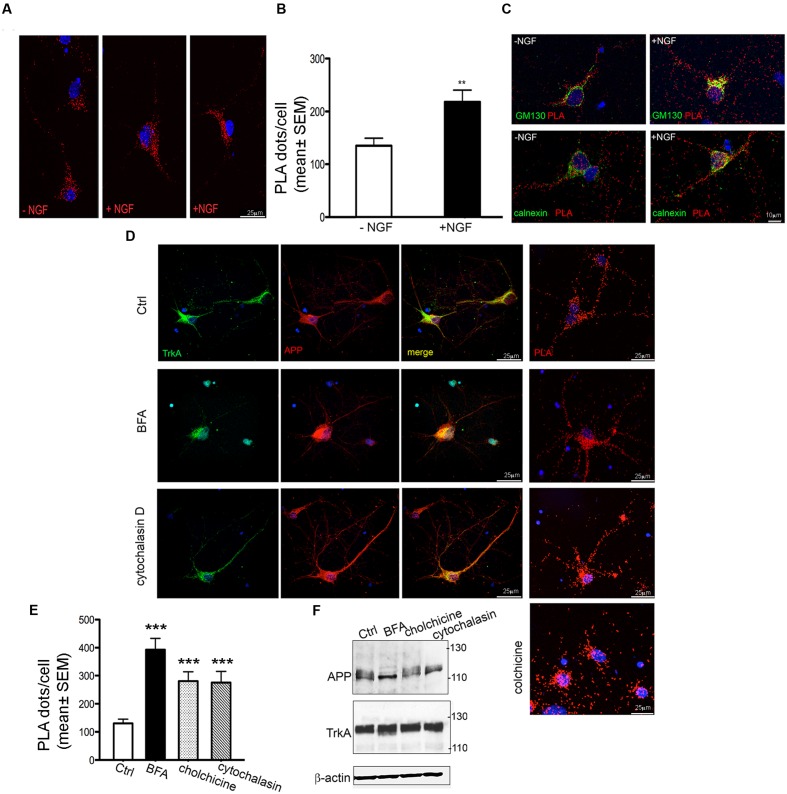
**Modulation of APP/TrkA association by NGF and trafficking perturbation.** **(A)** Representative images of PLA performed with APP-CT and Trk B3, antibodies showing the interaction of APP and TrkA in primary septal cultures (12 DIV) untreated (Ctrl) or treated with NGF (100 ng/ml for 1 h). Positive signal of interaction is shown as red dot, nuclei are stained with DAPI. **(B)** Quantification of the PLA signal in primary neurons untreated and treated with NGF (100 ng/ml for 1 h)). The graph represents quantitative analysis of the number of PLA fluorescent red puncta/cell counted in five different fields and for a total of 20–30 cells examined and are expressed as mean ± SEM ^∗∗^*p* < 0.01 Student *t*-test. **(C)** Co-localization of PLA signal with GM130 and calnexin in untreated and NGF treated cells. A representative z-stack of images was collected from each cell. **(D)** Confocal microscopy images of immunofluorescence and PLA signal [both performed with anti-APP-CT (red) and anti-Trk B3 (green) antibodies], of untreated (Ctrl) and BFA (5 μg/ml for 3 h), cytochalasin D (5 μM for 3 h) and colchicine (100 μM for 3 h) treated primary septal cultures (12 DIV) are shown. PLA dots in cytocalasin D treated cells are distributed in cell body and also along the neuritis coherently with the co-localization signal detected by immunofluorescence. Nuclei are stained with DAPI (blue). **(E)** Quantification of the PLA signal of APP/TrkA interaction in primary neurons untreated (Ctrl) and treated with BFA, cytochalasin D and colchicine. A total of 20–30 cells were examined. ^∗∗∗^*p* < 0.001 when compared to Ctrl. Student *t*-test was used for statistical analysis. **(F)** Representative Western blot analysis with anti APP-CT (A8717, Sigma Aldrich), anti TrkA-CT (Abcam, ab76291) antibodies and anti β-actin as loading control under treatments indicated.

Proximity ligation assay signals were prominent in the perinuclear region and along neuronal processes of primary neurons (**Figures 5C** and **6A**). By combining immunofluorescence with PLA assay, we found that PLA signal colocalizes with GM130 (*r* = 0.49 ± 0.18), a *cis* Golgi matrix protein, and with calnexin (*r* = 0.068 ± 0.049), an integral ER protein) (**Figure 6B**). Consistent with such a localization in ER and Golgi, APP/TrkA complexes in transfected HEK293 cells were shown to colocalize with ER and Golgi markers (mCherry ER-3 and tomato-mannosidase II protein respectively) as well as with marker of early endosome (Ds-red rab5) and recycling endosome (Ds-red rab-11) (**Supplementary Figure S5**).

Treatment with NGF (100 ng/ml for 1 h) increases the number of PLA dots by ∼50% compared to untreated cells (**Figures 6A,B**) thus confirming that NGF favors the interaction between TrkA and APP (Triaca et al., 2016). This increase occurred also in GM130 positive structures (**Figure 6C**) where the number of PLA dots colocalizing with GM130 was highly significantly increased in NGF treated cells compared to untreated cells (11724 ± 1478 and 6230 ± 1234 respectively; ^∗^*p* = 0.019 *t*-test). A similarly strong increase was observed in the number of PLA dots colocalazing with calnexin in NGF treated cells (14430 ± 2249 vs. 4602 ± 1302; *p* = 0.0316 *t*-test) (**Figure 6C**). Notably, the increased PLA signal under NGF treatment was not due to increased APP or TrkA levels (data not shown) as previously reported Triaca et al. (2016).

To confirm the colocalization of PLA signal with ER/Golgi, we performed PLA assay after application of brefeldin A (BFA, 5 μg/ml for 3 h) a macrocyclic lactone antibiotic that induces redistribution of Golgi proteins into the ER and blocks anterograde transport (Pelham, 1991). Indeed, in BFA treated neurons APP and TrkA co-localized in the entire cell body and the initial segment of neurites, being absent in the distal part of neurites. Accordingly, the PLA signal was intense throughout the entire cell body and the initial segment of the neurites (**Figure 6D**).

The overall effect of BFA on TrkA/APP interaction was further determined by counting the number of dots per cell. Under BFA treatment the number of complexes increased by 3.5 fold compared to untreated cells (**Figure 6E**).

Similar results were obtained by disrupting microtubule-dependent anterograde and retrograde transport of vesicles (Cid-Arregui et al., 1995; Bloom and Goldstein, 1998) within the neurites with the use of colchicine (100 μM for 3 h) (LeBlanc and Goodyer, 1999) and by perturbing the actin cytoskeleton and the endocytic pathway with cytochalasin D (5 μM for 3 h) (Fujimoto et al., 2000) (**Figures 6D,E**). Quantification of PLA dots showed that colchicine treatment increased by twofold the number of interaction events. At the dose used, BFA, cytochalasin D and colchicine had no effect on APP and TrkA levels (**Figure 6F**) and on cell survival (data not shown). Overall, these experiments confirm the specificity of TrkA/APP localization at Golgi and ER sites.

### Decrease of TrkA/APP Complex during Cell Death

**FIGURE 7:**
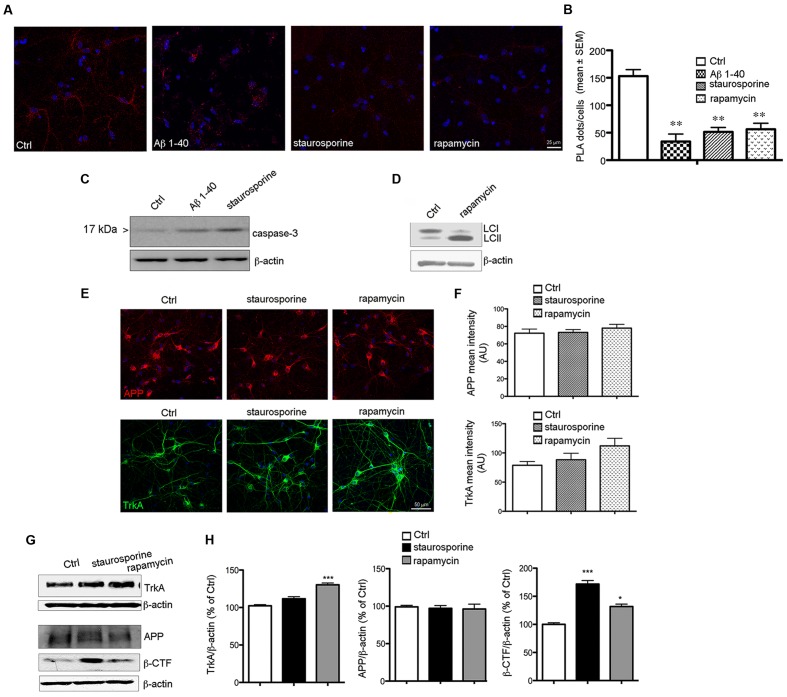
**Reduction of PLA signal during cell death.** **(A)** Representative PLA assay performed with APP-CT and Trk B3 antibodies in primary septal cultures (12 DIV) untreated (Ctrl) or treated with Aβ 1–40 (20 μM), staurosporine (30 nM) and rapamycin (10 nM) for 6 h. **(B)** The graph represents quantitative analysis of the number of PLA fluorescent red puncta/cell counted in five different fields and for a total of 20–30 cells examined and are expressed as mean ± SEM (^∗∗^*p* < 0.005, Student *t*-test was used for statistical analysis). **(C)** Western blot analysis for cleaved and active capsase-3, is reported for Ctrl, Aβ 1–40 and staurosporine treated cells for 12 h. **(D)** Representative blot for LC3 in Ctrl and rapamycin treated cells for 12 h. Post-translational modification of cytosolic LC3-I (Light Chain 3 of Microtubule Associated Protein 1A/B) to LC3-II is a useful index of autophagy. **(E)** APP and TrkA expression in Ctrl and staurosporine and rapamycin treated cells. Double immunofluorescence for APP (red) or TrkA (green) and DNA (DAPI, blue) in Ctrl condition and following the indicated treatments for 12 h. **(F)** Bar plot summarizes the APP and TrkA mean fluorescence intensity in Ctrl and treated cultures. Fluorescence intensity is reported in arbitrary unit (AU). No significant difference were observed between Ctrl condition and following staurosporine or rapamycin exposure. **(G)** Representative Western blot of TrkA, APP and β-CTF in neurons treated with staurosporine (30 nM) and rapamycin (10 nM) for 12 h. **(H)** Graph depicting quantification of immunoreactivity for TrkA, APP and β-CTF bands normalized to β-actin (used as loading control) in neurons treated with staurosporine and rapamycin for 12 h (^∗^*p* < 0.05; ^∗∗∗^*p* < 0.0001). Results are the means (±SEM) of duplicate determinations from three independent experiments and are reported as percentage of Ctrl cells.

We have recently demonstrated that the amount of TrkA bound to APP is reduced in AD brains (Triaca et al., 2016). Since neuronal loss is a pathological feature of AD, we investigated the fate of TrkA/APP complex in different paradigm of cell death. Aβ-peptide has been reported to induce apoptosis on cultured primary neurons (Shaked et al., 2006). We found that TrkA/APP interaction was dramatically reduced 12 h following exposure to 20 μM Aβ-peptide 1–40 (22 ± 9% compared to Ctrl untreated cells: ^∗∗^*p* = 0.005 *t*-test) (**Figures 7A,B**). Importantly, this decrease in APP/TrkA complexes was apparent before overt signs of cell death. Indeed, viability detected by MTT assay and intact nuclei count was not significantly different between treated and untreated cultured cells (**Supplementary Figure S6**) and the active caspase-3 was slightly increased in Aβ-treated cells compared to control (**Figure 7C**), showing that disruption of TrkA/APP complexes precedes Aβ-peptide mediated cell death. Dispersal of TrkA/APP interaction was also observed with more aggressive and toxic Aβ-peptide 1–42 and 25–35 (**Supplementary Figure S7**). Similarly, we found that other pro-death agents such staurosporine (30 nM) which induces apoptosis with caspase-3 activation (**Figure 7C**) and rapamycin (10 nM), which causes autophagy (**Figure 7D**) induced a disruption of TrkA/APP complexes observable within 6 h (PLA dots/cell: 51 ± 6 and 57 ± 8 respectively compared to Ctrl untreated cells) (**Figures 7A,B**) when viability was not significantly affected (**Supplementary Figure S6**).

Notably, we found that the staining level for APP and TrkA was similar in Ctrl, staurosporine and rapamycin treated cells suggesting that the decrease of TrkA/APP complex is not caused by their degradation (**Figures 7E,F**). Moreover, no decrease in the level APP or TrkA were observed by WB under staurosporine and rapamycin treatments (**Figure 7G**)

Interestingly, we found that loss of TrkA/APP interaction was accompanied with an increased production of β-CTF APP fragment (**Figures 7G,H**). These data demonstrate that TrkA/APP complexes are sensitive to cell death stimuli associated with amyloidogenic APP processing, which also occurs following NGF deprivation (Matrone et al., 2008).

## Discussion

Formation of TrkA/APP complexes is correlated with the physiology and pathophysiology of APP processing and transcriptional activity (Tarr et al., 2002a; Zhang et al., 2014; Triaca et al., 2016) and NGF-TrkA signaling (Matrone et al., 2011; Zhang et al., 2013). However, no evidence of a close proximity of the two proteins has been documented prior to this work. Resolving this issue is relevant to resolving the physiological and pathological roles of APP, a duality that hinges in CBFN on the supply of NGF. Therefore, we used co-immunoprecipitation, BiFC and PLA to get detailed insight into the formation of TrkA/APP complexes. In the field of neurodegenerative diseases, BiFC and PLA have frequently been used to study protein-protein interactions in Alzheimer’s and Parkinson’s disease (Chen et al., 2006; Waldron et al., 2008; Gonçalves et al., 2010; Isbert et al., 2011; Weyer et al., 2011; Teranishi et al., 2012; Gu et al., 2013; Riese et al., 2013; Lundgren et al., 2015; Roberts et al., 2015). Both BiFC and PLA can be used to test whether two proteins are in close proximity, although do not specify if they bind directly or if their interaction is mediated by a common partner.

By co-immunoprecipitating TrkA with APP deletion mutants, we identified APP residues 597–612 as essential for binding to TrkA (**Figure 1B** and see also Zhang et al., 2014), which we confirmed by BiFC assay (**Figures 3D,E,G**). Additionally, we showed that TMD/intracellular domains of TrkA are involved in the interaction with APP (**Figure 1D**), and that the region of TrkA residues 354–488 is sufficient for binding with APP (**Figures 3H,I**). Taken together these data point to the transmembrane/juxtamembrane domains of TrkA and APP as crucial for TrkA/APP complex formation. Interestingly, previous studies (Fombonne et al., 2009) demonstrated that the same residues in APP (597–612) are also important for binding to p75NTR. This short stretch of amino acids contains the α and β secretase recognition sites and is therefore essential for the production of either physiologically relevant APP processed forms or toxic fragments such as Aβ peptides and β-CTF. We therefore suggest that competition between p75NTR and TrkA for association with APP through this short stretch of amino acids in APP may underlie the inhibition of TrkA/APP complex formation by p75NTR. In addition, competition for binding to the same surface may explain the functional antagonism of the two NGF receptors, with p75NTR promoting and TrkA reducing the formation of Aβ peptides and β-CTF (Rossner et al., 1998; Fombonne et al., 2009; Zhang et al., 2014; Triaca et al., 2016). By the same reasoning, it is tempting to speculate that TrkA binding also reduces formation of APP homodimers, an interaction that requires the APP transmembrane domain and correlates with amyloidogenic processing of APP. Interestingly, we showed that NGF, which protects neurons from cell death and the amyloidogenic processing of APP, also increases TrkA/APP association (**Figure 6A**). Importantly, APP homodimer formation has many physiological functions. For instance, homodimerization involving the APP ectodomain supports the role of APP in neuronal migration during embryogenesis (Young-Pearse et al., 2007) and homodimerization involving the APP transmembrane domain is important for APP processing by β and γ secretases (Scheuermann et al., 2001).

We have also shown that p75NTR, Mint2 and ShcC, three partners of both APP and TrkA, reduce rather than favor TrkA/APP complex formation (**Figures 4A,B**). It would be important to assess whether p75NTR, Mint2 or ShcC contribute to regulating APP homodimerization.

The TrkA/APP complexes were found in the ER, Golgi, cell surface and endocytic vesicles. This distribution, shown by co-localization with compartment-specific markers (**Figures 2C** and **6C**; **Supplementary Figure S5**) was confirmed by treatment with drugs that perturb cellular trafficking (**Figure 6D**) and is consistent with the known physiological trafficking of APP and TrkA (Haass et al., 2012; Goh and Sorkin, 2013; Farina et al., 2015). Both APP and TrkA form homodimers in the ER/Golgi before reaching the cell surface (Khalifa et al., 2010; Shen and Maruyama, 2011; Farina et al., 2015).

TrkA spontaneously dimerizes in the ER before reaching the cell surface, where it has full accessibility to NGF. NGF binding induces the rotation of the transmembrane domains of the preformed receptor dimers, resulting in rearrangement of the cytoplasmic domains for activation and transmission of NGF-mediated signaling cascades (Shen and Maruyama, 2011). Thus, it is likely that the known effect of APP in regulating the NGF/TrkA signaling pathway (Matrone et al., 14; Shen and Maruyama, 2011; Zhang et al., 2013) might require a fine regulation of balance between TrkA dimer and TrkA/APP complexes. Importantly, NGF increases the number of TrkA/APP complexes in every compartment, including the ER and Golgi apparatus, as measured by PLA on rat primary septal neurons (**Figures 7B,C**). This increase in TrkA/APP complexes did not depend on augmented levels of TrkA and APP, and occurred within 1 h of treatment with NGF. Although we do not yet know the signaling pathway that is responsible for this effect, a possible explanation may come from some of our recent findings. In particular, phosphorylation of APP Thr668 prevents the interaction between TrkA and APP, and NGF stimulation decreases APP phosphorylation in Thr668, via inhibition of c-Jun N-terminal protein kinase (JNK) (Triaca et al., 2016).

We observed that TrkA/APP complexes decrease in number, without apparent TrkA or APP degradation, as an early response to cell death stimuli such as Aβ peptide, staurosporine or rapamycin. Dissociation of TrkA/APP complexes is clearly observable before loss of cell viability (**Figure 7A**; **Supplementary Figure S6**). Although the cascade of events that lead to cell death upon these treatments may differ, it is intriguing that both Aβ and staurosporine rely on increased APP/APP dimer formation, which leads to increased production of Aβ peptide via a positive feedback mechanism. In fact, APP/Aβ-peptide binding induces APP homodimerization, which may, in turn, influence Aβ production (Scheuermann et al., 2001; Shaked et al., 2006; Munter et al., 2007; So et al., 2012). On the other hand, staurosporine-induced cell death, which is characterized by amyloidogenic APP processing (Sodhi et al., 2008) could be due to the up-regulation of APP phosphorylation at T668 (Sodhi et al., 2008). If we assume that TrkA bound to APP prevents APP homodimerization, the dissociation of TrkA/APP complexes would be an early event necessary to allow formation of APP/APP complexes. Further studies are necessary to assess the interplay and the relevance of these phenomena.

Overall, our finding that a 16 amino acid stretch of APP contains the determinants not only for its physiological as well as pathological processing, but also for their modulation by the TrkA receptor, focuses new attention on the interactions between APP and TrkA and the possibilities for modulating their reciprocal interplay. This is especially true given the known importance of TrkA in translating crucial messages during development and in the adult brain.

## Author Contributions

NC designed experiments, performed PLA assay, acquired images, analyzed data, supervised and wrote paper; IP participated in cloning, transfection, data collection related to BiFC and co-immunoprecipitation, LL participated in the initial method optimization for investigating TrkA/APP interaction; MC and CP participated in method optimization and primary septal cultures establishment and treatments; MP and FB participated in method optimization of FACS analysis; VT and VS provided reagents and expert opinions on TrkA and APP; DM contributed reagents; IM, provided TrkA-VC/VN and ErbB3 plasmids; AL provided expert opinions on TrkA and critically revised the manuscript. PC supervised and funded the project. All authors reviewed the manuscript.

## Conflict of Interest Statement

The authors declare that the research was conducted in the absence of any commercial or financial relationships that could be construed as a potential conflict of interest.

The reviewer PL and handling Editor declared their shared affiliation, and the handling Editor states that the process nevertheless met the standards of a fair and objective review.

## Footnotes

1 http://rsb.info.nih.gov/ij
