# Supplementary figures and images for: Association of TrkA and APP Is Promoted by NGF and Reduced by Cell Death-Promoting Agents
Source: Front Mol Neurosci. 2017 Jan 31;10:15. doi: 10.3389/fnmol.2017.00015 (PMC5281621; doi:10.3389/fnmol.2017.00015)

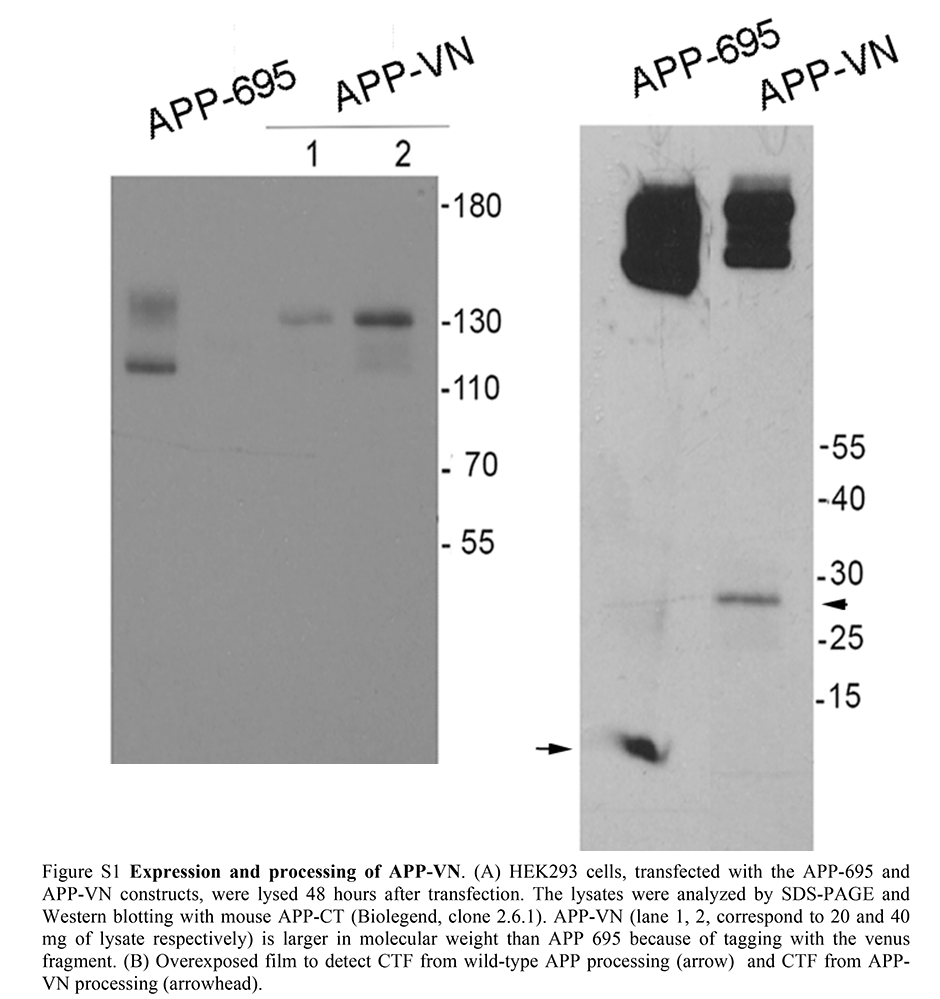

Supplement: Supplementary file 1 [file Image_1.TIF]

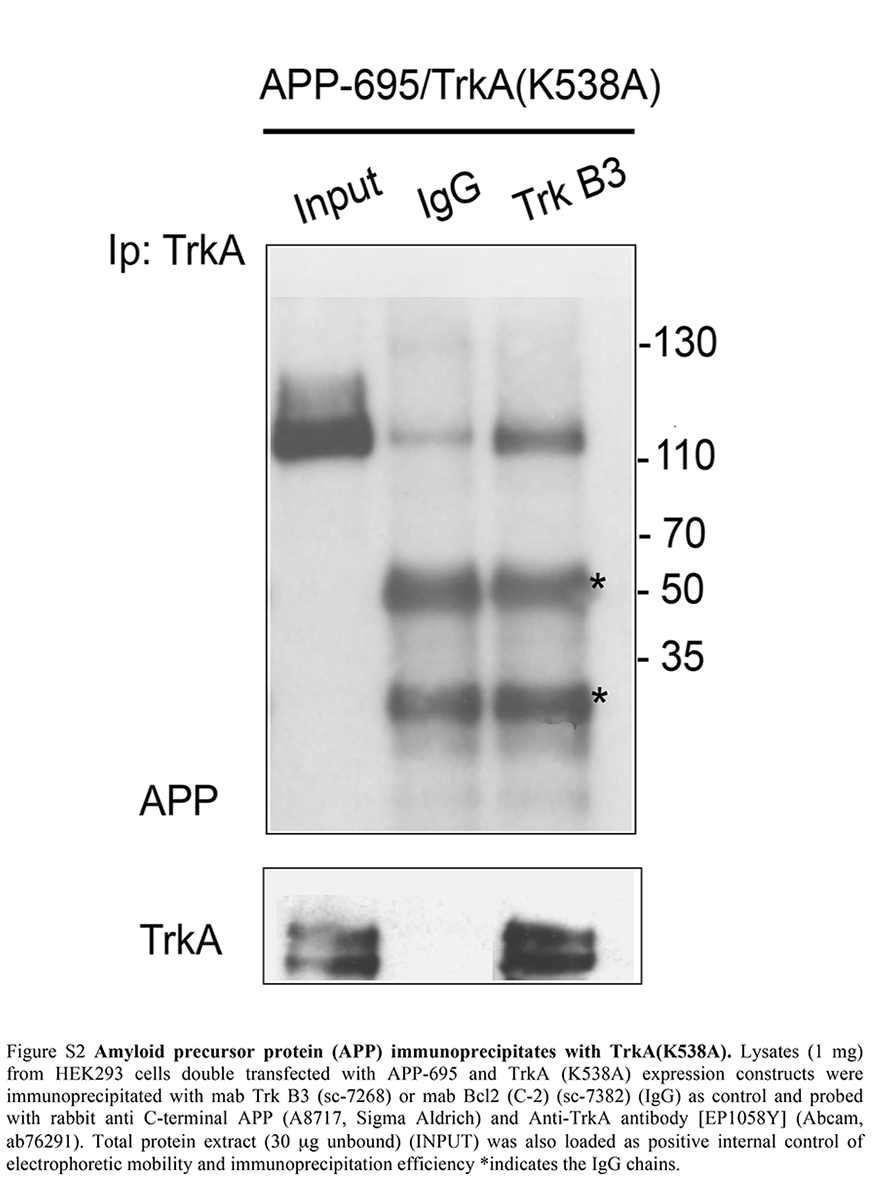

Supplement: Supplementary file 2 [file Image_2.TIF]

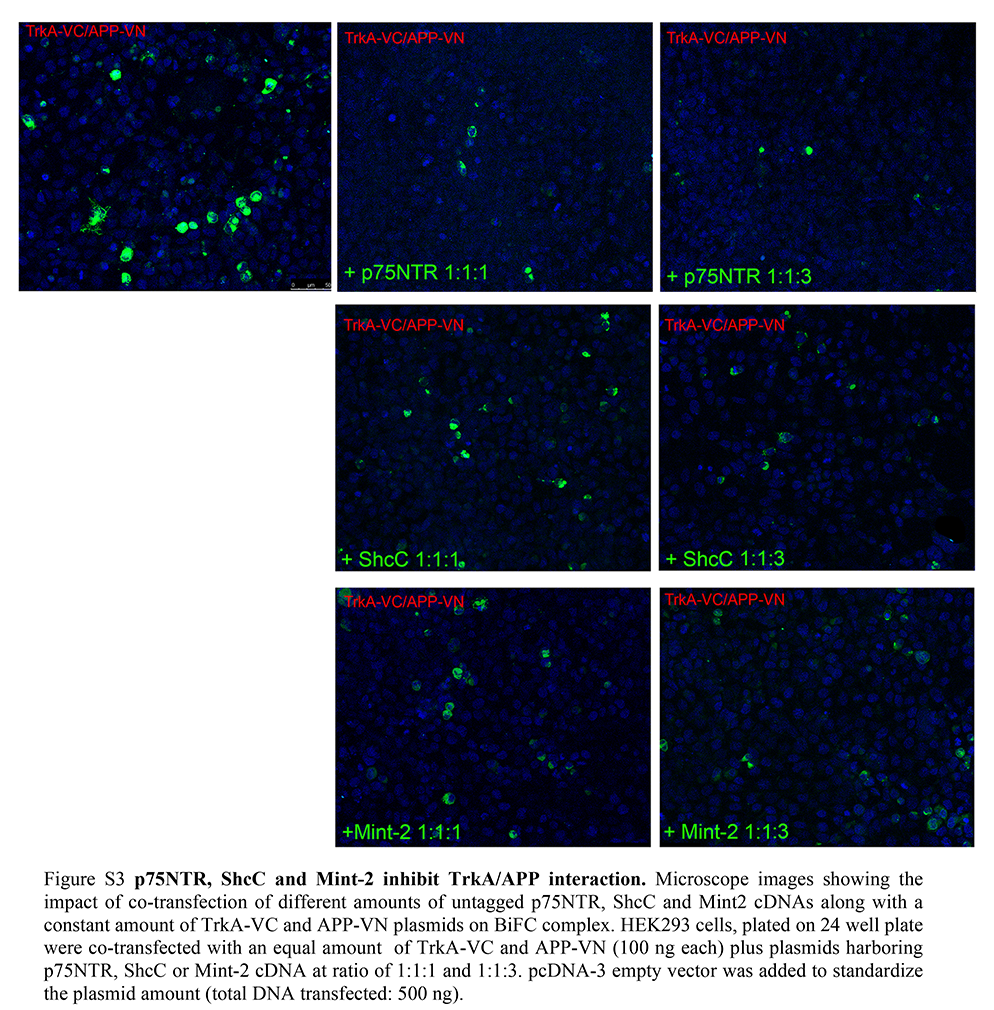

Supplement: Supplementary file 3 [file Image_3.TIF]

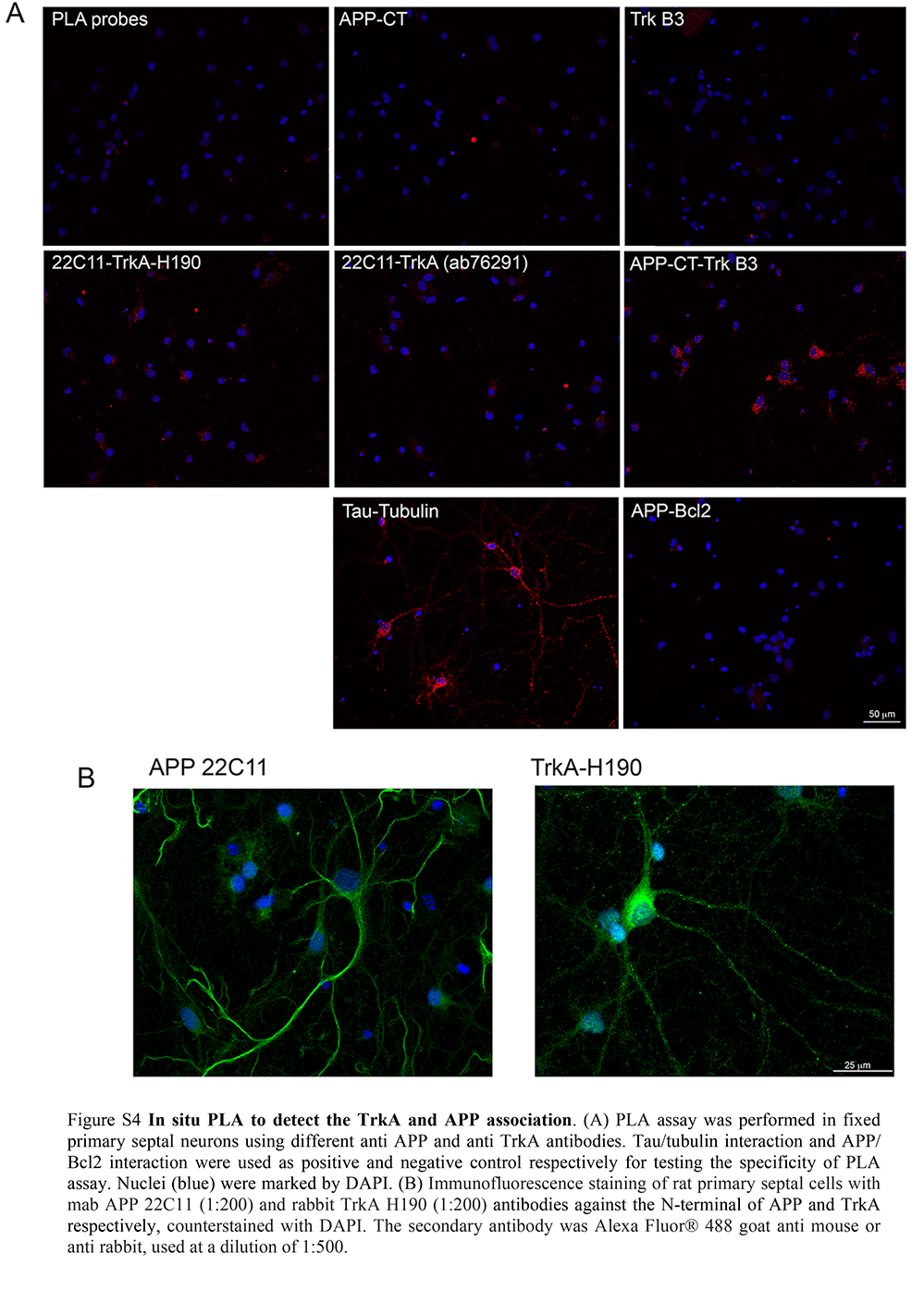

Supplement: Supplementary file 4 [file Image_4.TIF]

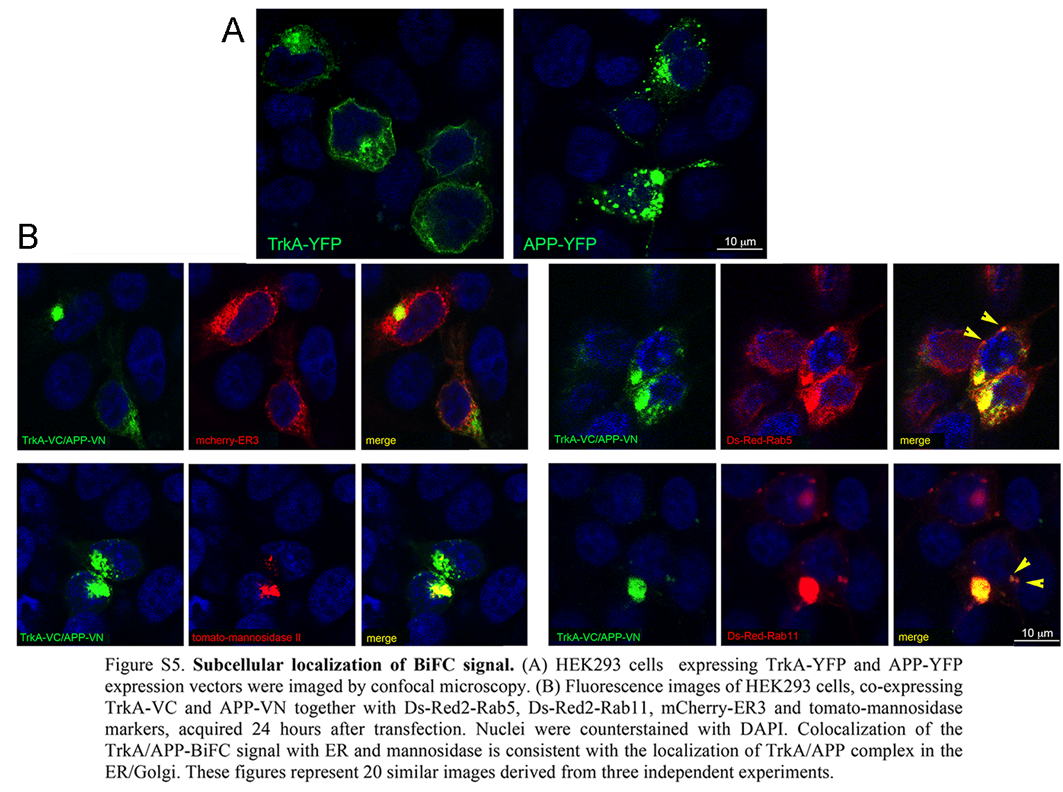

Supplement: Supplementary file 5 [file Image_5.TIF]

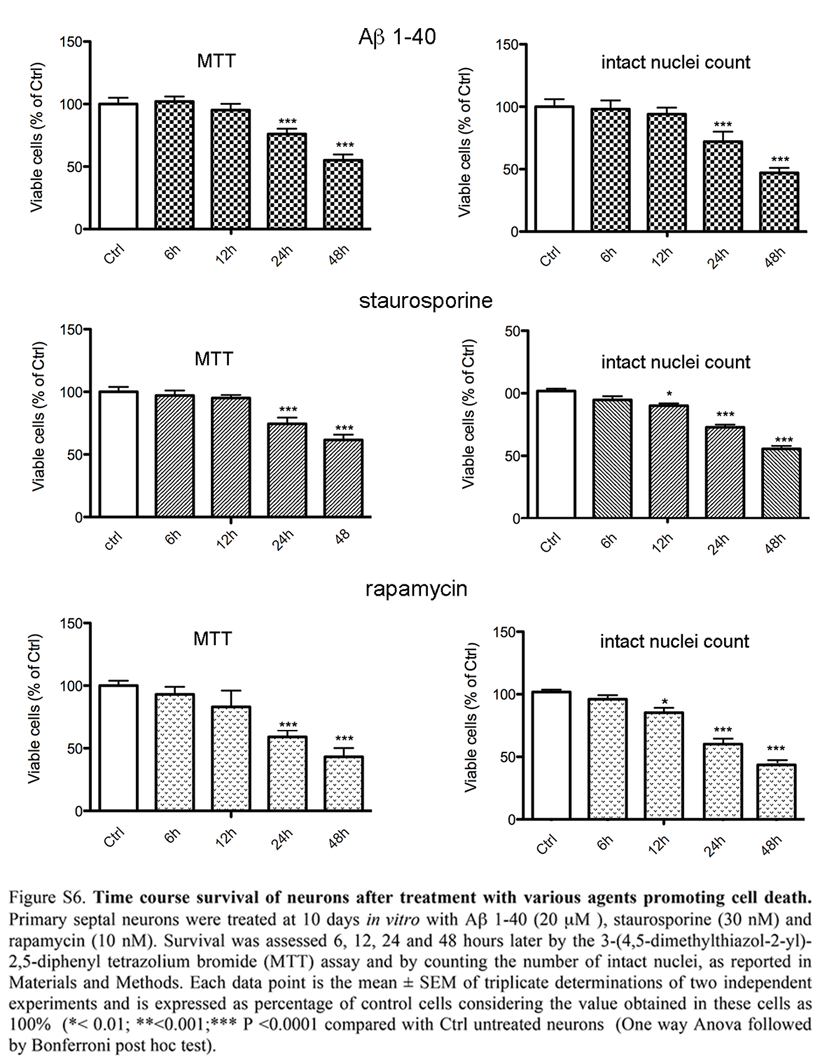

Supplement: Supplementary file 6 [file Image_6.TIF]

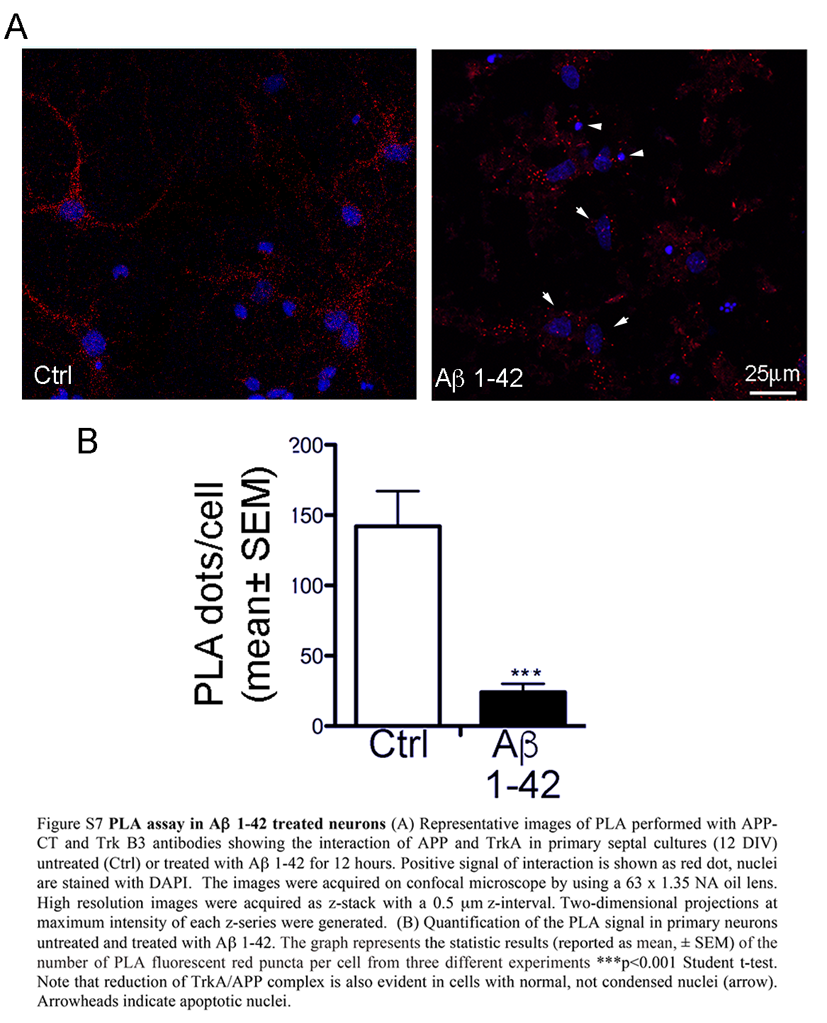

Supplement: Supplementary file 7 [file Image_7.TIF]
